# Knowledge, perceptions and confidence of physicians and pharmacists towards pharmacogenetics practice in Kuwait

**DOI:** 10.1371/journal.pone.0203033

**Published:** 2018-09-05

**Authors:** Abdullah Albassam, Shahad Alshammari, Ghadeer Ouda, Samuel Koshy, Abdelmoneim Awad

**Affiliations:** 1 Department of Pharmacy Practice, Faculty of Pharmacy, Kuwait University, Kuwait City, Kuwait; 2 Department of Pharmacy, Jahra Hospital, Ministry of Health, Jahra City, Kuwait; 3 Drug Inspection Administration, Ministry of Health, Kuwait City, Kuwait; Drake University College of Pharmacy and Heath Sciences, UNITED STATES

## Abstract

**Background:**

Pharmacogenetics practice has been successfully implemented in many developed countries to enhance personalized medicine and improve clinical and economic outcomes. An understanding of healthcare providers’ knowledge, perceptions, confidence towards pharmacogenetics, and their active enrollment with pharmacogenetic testing is essential for test acceptance and utilization. This study was designed to assess physicians’ and pharmacists’ knowledge, perceptions, and confidence towards pharmacogenetics, determine the preferred learning format for their future education in pharmacogenetics, and identify the barriers to its application in their practice settings.

**Methods:**

A cross-sectional survey was conducted using a pretested self-administered questionnaire on a sample of 629 randomly selected physicians and pharmacists. Descriptive and comparative analyses were used in data analysis.

**Results:**

The response rate was 98.1%. Less than one-tenth of respondents were exposed to pharmacogenetics education or training (8.9%), applied pharmacogenetics testing in their practice (9.4%), or provided patient counselling on the results of the pharmacogenetic testing (9.1%), and over 90% of them were physicians. The overall respondents’ mean (SD) total knowledge score percentage was low [45.0% (24)] and there was no significant difference between the physicians and pharmacists scores (p>0.05). Only 16.0% of participants indicated that they felt confident in applying pharmacogenetics in their practice settings. Despite these low levels of knowledge and self-confidence, 70.2% of participants expressed overall positive perceptions towards pharmacogenetics and its clinical implications. These positive overall perceptions were found to be significantly more common among pharmacists compared to physicians (p<0.05). The top two perceived barriers facing the implementation of pharmacogenetics in Kuwait were lack of education or training and clinical guidelines.

**Conclusions:**

These findings highlight important concerns and will aid in the assessment of current pharmacogenetics practice. Also, they will provide further insight in designing future targeted multifaceted interventions to promote the adoption and utilization of pharmacogenetics testing in Kuwait.

## Introduction

Pharmacogenetics is defined as *“the study of how genes affect a person’s response to drugs*. *It combines pharmacology (the science of drugs) and genomics (the study of genes and their functions) to develop effective*, *safe medications and doses that will be tailored to a person’s genetic makeup*” [1]. In the United States of America (USA), approximately 750 million prescriptions for pharmacogenetically high-risk medications were prescribed in 2013, and it is estimated that 99% of the population has high-risk variant for a gene associated with these drugs [2]. There is scarcity of data to comprehend the patterns of pharmacogenetic variants in the Arab populations. Recent studies reported the distinct occurrence of genetic variants associated with warfarin dosage in Kuwaiti population [3,4].Other studies described the pharmacogenetic maps of variants associated with warfarin and clopidogrel in Qatari and Omani population [5,6]. Hence, prediction of the probability of drug efficacy or potentially harmful adverse events, based on the outcomes of pharmacogenetics testing, will help clinicians to enhance personalized medicine and improve clinical and economic outcomes [7].

Pharmacogenetics testing is currently available to guide and support better treatment decisions for some patients. Relevant examples of the impact of pharmacogenomics include (1) use of HLA-B*1502 genotyping before the initiation of carbamazepine therapy, to prevent Steven Johnson syndrome and toxic epidermal necrolysis; (2) use of HLA-B*5701 genotyping prior to initiation of abacavir therapy, to avoid serious hypersensitivity syndrome; (3) use of CYP2C19 genotyping before the initiation of clopidogrel therapy, to reduce risk of cardiovascular events; and (4) use of VKORC1/CYP2C9 genotyping prior to initiation of warfarin therapy, to assist in determining the appropriate initial dose of warfarin and preventing haemorrhagic incidents [8–10]. The impact and clinical utility of pharmacogenetics testing is exemplified and further strengthened by the requirements of the United States’ Food and Drug Administration (US FDA), to include human genomic information in over 100 medications labels recommending patient-specific dosing strategies [11]. Despite the availability of pharmacogenetic tests, their clinical application in patient care is reported to be slow. Previous studies have identified major barriers to healthcare providers’ acceptance of pharmacogenetics testing into their practice. These barriers included lack of knowledge, awareness and confidence among healthcare professionals in implementing pharmacogenetic information within patient care [7,12]. Hence, the provision of enhanced genetic education to healthcare professionals at both the undergraduate and in-service levels is of paramount importance to the implementation of pharmacogenetics testing in clinical practice. Accreditation and educational bodies have recently started to include pharmacogenetics in their facilities’ curricula [13,14]. Other challenges also need to be overcome such as the perceived lack of evidence for the clinical benefits of pharmacogenetics testing, limited resources, and lack of availability of the tests [15]. A considerable number of these medications such as such as warfarin, clopidogrel, statins, glibenclamide, gliclazide, glimepiride, allopurinol, opioid analgesics, selected psychotropic medications, kidney transplant medications, and cancer medications are commonly used in Kuwait. The Molecular Laboratory in Kuwait Cancer Control Center currently provides the pharmacogenetics testing service for 52 medications. Pharmacists, as healthcare providers and medication experts regularly interact with other healthcare professionals and patients to ensure effective and safe medication therapy. Therefore, they have the potential to play a crucial role regarding dose adjustments and/ or medication choice based on the outcomes of pharmacogenetic testing. Also, they can provide pharmacogenetic counselling to enable patients and healthcare providers to understand and utilize pharmacogenetic information, for better therapeutic outcomes [16]. The pharmacist’s role in leading interprofessional collaboration for the development of guidelines and the initiation of pharmacogenetic services has recently been highlighted by the American Society of Health-System Pharmacists [17]. Pharmacist-lead pharmacogenetic programs have already been introduced in various institutions [17,18].

Several studies have been performed worldwide to assess healthcare providers’ knowledge, awareness, responsibilities and attitudes towards pharmacogenetics, and their educational needs for the implementation of pharmacogenetic testing-based treatment options. Most of these studies were conducted in the USA [7,16,19–22] and few studies have been performed in Canada, Australia, Europe and Africa [16,23]. Yet, little is known about similar outcomes in the Eastern Mediterranean region: to date, only one study has been published, in Qatar [24]. Due to the positive and evident impact of pharmacogenetics testing in clinical and economic outcomes, there is a need to address healthcare providers’ knowledge, perceptions and confidence towards pharmacogenetics and its implementation, and to tailor such research further for the local context. However, although many developed countries have successfully implemented the clinical practice of pharmacogenetics to enhance the concept of personalised medicine and improve the quality of health practice, there are no published studies to date to understand the current status of pharmacogenetic practice in Kuwait. Hence, this study was designed to assess physicians’ and pharmacists’ knowledge, perceptions, and confidence towards pharmacogenetics, determine the preferred learning format for their future education in pharmacogenetics, and identify the barriers to its application in their practice settings.

## Materials and methods

### Study design and population

A descriptive, cross-sectional survey was conducted in Kuwait, a Middle-Eastern country with an area of 17,820 km^2^ and an estimated population of 3,892000 people (2015 estimate) [25]. It was conducted during the period from January to April 2016. The study population included all physicians and pharmacists working in the six general public hospitals in Kuwait. Ethical approval for this study was obtained from the Ministry of Health Ethical Committee, Kuwait.

Physicians and pharmacists working in healthcare settings in Kuwait have diverse educational backgrounds, with education and training from Kuwait, other Middle Eastern countries, as well as other countries such as the United States of America, Canada, United Kingdom, and India. Faculty of Medicine and Faculty of Pharmacy at Kuwait University are the only medical faculties in Kuwait. Medical students receive their formal education over seven years and graduate with the Doctor of Medicine (MD) degree. Students in the faculty of pharmacy complete their formal education over five years and graduate with a Bachelor of Pharmacy (BPharm) degree. The undergraduate pharmacy curriculum is designed to develop students’ professional abilities to make rational and evidence-based clinical decisions. Pharmacy students undertake experiential training (clerkships) supervised by professional pharmacy and medical preceptors in various healthcare settings during the fourth and fifth years of their study. In 2016, the Faculty of Pharmacy started a two-year add-on Doctor of Pharmacy (PharmD) program to provide advanced clinical skills and practice experiences to provide optimal clinical pharmacy services to patients. Pharmacogenomics is currently taught with limited depth over few hours in the undergraduate curricula for pharmacy and medical schools in biochemistry and pharmacology courses, which is inadequate in comparison to USA and other universities in developed countries.

The sample size was based on the assumption that the proportion of responses to most of the main questions would be 50%, due to the fact there are no previous similar studies from Kuwait. It was determined using the Raosoft sample size calculator using a margin of error of 5% and a confidence interval of 95%, for a target population size of 1464 physicians and 403 pharmacists currently practicing in the six hospitals [26]. The minimum sample size estimated for the study was 306 physicians and 197 pharmacists. Assuming a response rate of 80%, a sample size of 383 physicians and 246 pharmacists were enrolled in the survey. They were randomly selected from the six hospitals using stratified and systematic random sampling according to the methodology described by the World Health Organization [27].

The selected physicians and pharmacists were contacted and provided with an explanation about the aim of the study. They were free to decline to participate. Data were collected anonymously via a self-administered survey. Those who agreed to participate in the study were given the questionnaires, which were completed anonymously and collected within 1–2 weeks. They were assured of confidentiality and gave written consent to contribute to the study.

### Study questionnaire

Based on a literature review of similar previous studies, the study survey was adapted from validated questionnaires that were previously used in the USA and Qatar [7,19,24]. The content validity of the adapted questionnaire was established by a research group at Kuwait University. Face validity of the survey was assessed with five pharmacists and five physicians for clarity of questions. Then the survey was pretested for content, design, readability, and comprehension on five pharmacists and five physicians, and suitable amendments were made so that the questionnaire was simple to understand and answer, yet gave accurate data.

The pre-tested questionnaire consisted of six sections and contained both open-ended and close-ended questions. The first section included six items to provide information about the demographic and professional characteristics of respondents. Section two consisted of three questions to provide information about respondents’ previous exposure to pharmacogenetic training or education, current application of pharmacogenetic testing in their practice settings, and the provision of patient counselling on the results of this pharmacogenetic testing. Respondents were asked to answer these questions by choosing ‘yes’ or ‘no’. The third section included five items to assess the participants’ general knowledge on pharmacogenetics. One of these items is on the package insert for warfarin and whether it included pharmacogenomics information. The label on the warfarin package insert available in Kuwait is similar to the US FDA label for warfarin. The responses to these items were either ‘true’, ‘false’ or ‘do not know’. Section four included six items to determine the respondents’ perceptions towards pharmacogenetics and its application in their clinical practice. The fifth section included four items to identify the participants’ confidence in applying pharmacogenetics in their practice settings. For ease of interpretation and analysis of the results, the responses for the perceptions and confidence sections were measured using a three-point Likert scale (agree, neutral, disagree). Section six included a list of learning formats for future education in pharmacogenetics, of which respondents were allowed to choose one or more, as well as an option to recommend any format not included in the list. The last section provided a list of barriers that might hinder the application of pharmacogenetic testing in their practice settings. Participants were allowed to choose one or more, as well as an option to add any barrier not included in the list. The items of the questionnaire and their quantified responses are presented in tables and figures in the results section.

### Statistical analysis

Data were analysed using the Statistical Package for Social Sciences (IBM SPSS Statistics for Windows, version 23, Armonk, NY: IBM Corp). The study participants’ responses were presented as percentages (95% confidence intervals; CI) and means (standard deviation-SD). In relation to the five items for assessing knowledge, a score of one point was given if the right answer was chosen and a score of zero was given if the wrong answer or ‘do not know’ was chosen. The percentage knowledge score (PKS) was calculated by dividing the participant’s score by 5 (the maximum score) and multiplying by 100. PKS was expressed as mean (SD). The internal consistency for the sections to assess the respondents’ self- perceptions and confidence towards pharmacogenetic was assessed using Cronbach’s α test. The test results were as follows: six items for perceptions (0.77) and four items for confidence (0.82). The perceptions score was calculated as a continuous variable by summing the participant’s number of appropriate responses to 6 statements. One point was awarded for each appropriate response (agree) and zero for each disagree or neutral response, with a maximum obtainable score of 6 for each respondent. The perceptions score was categorized into two levels indicated by negative (0–3) and positive (4–6). The confidence score was calculated similarly, with a maximum obtainable score of 4 for each respondent. The confidence score was categorized into two levels indicated by low (0–2) and high (3–4). The perceptions and confidence scores were reported as mean (SD). The Mann–Whitney test was used to evaluate the differences in the overall scores between two groups of independent variables (profession: physicians vs. pharmacists; age: ≤ 35 years vs. ≥ 36 years; gender: males vs. females; practice experience: ≤ 10 years vs. > 10 years; and previous pharmacogenetic training or education: yes vs. no). Data from the responses to evaluate perceptions and confidence were compared between the physicians and pharmacists using chi-square tests. Statistical significance was accepted at a p value < 0.05.

## Results

A total of 629 healthcare providers were approached to participate in the study, 617 of whom agreed and completed the survey (a response rate of 98.1%). Of the respondents, 379 (61.4%) were physicians and 238 (38.6%) were pharmacists. The majority of pharmacists were inpatient pharmacists (58.0%), and the majority of physicians were resident physicians (46.0%). The mean (SD) age and experience as practitioners of the study population were 35 (9) years and 11 (9) years, respectively. Table 1 presents the respondents’ characteristics and professional information.

**Table 1:** Respondents’ demographic characteristics and professional information (n = 617).

Variable	Pharmacists n = 238	Physicians n = 379	Total n = 617
	n (%)	n (%)	n (%)
Age*			
≤35 years	138 (58.0)	239 (63.1)	377 (61.1)
≥36 years	89 (37.4)	139 (36.7)	228 (37.0)
***Gender***			
Male	94 (39.5)	231 (61.0)	325 (52.7)
Female	144 (60.5)	148 (39.0)	292 (47.3)
Experience*			
< 10 years	117 (49.2)	198 (52.2)	315 (51.1)
≥10 years	113 (47.5)	180 (47.5)	293 (47.5)
***Postgraduate study (Post-professional degree)***			
Yes	31 (13.0)	178 (47.0)	209 (33.9)
No	207 (87.0)	201 (53.0)	408 (66.1)

* Percentage may not total 100% due to some missing responses

Fewer than one-tenth of the respondents indicated that they had previous pharmacogenetic training or education (n = 55; 8.9%; 95% CI: 6.8–11.5); applied pharmacogenetics testing in their practice settings (n = 58; 9.4%; 95% CI: 7.3–12.1], and provided patient counselling on the results of the pharmacogenetics testing (n = 56; 9.1%; 95% CI: 7.0–11.7). Over 90% of these respondents were physicians.

### Respondents’ level of knowledge about pharmacogenetics

The overall respondents’ mean (SD) PKS was low [45.0% (24)]: 50.2% (n = 310; 95% CI:46.2–54.3) answered one to two items correctly [low knowledge = < 60%], 36.5% (n = 225; 95% CI: 32.7–40.4) answered three items correctly [moderate knowledge = 60%]; 13.3% (n = 82; 95% CI:10.8–16.3) answered four to five items correctly [high knowledge = ≥ 80%]. Only 1.6 (n = 10; 95% CI: 0.8–3.1) answered all five items correctly. Table 2 presents the participants’ responses to the five items used to assess their knowledge on pharmacogenetics. Fewer than one-fifth (n = 100; 16.2%; 95% CI: 13.4–19.4) of respondents recognised that genetic determinants of drug responses do not change over a person’s lifetime. Approximately three-quarters (n = 453; 73.4%; 95% CI: 69.7–76.8) of participants were aware that pharmacogenetics has an important role in individualizing response to medications.

**Table 2:** Respondents knowledge about pharmacogenetics (n = 617).

Items assessing knowledge	Correct answer	Answering correctly n(%; 95% CI)	Answering incorrectly n(%; 95% CI)	Answering “do not know” n(%; 95 CI)
Genetic determinants of drug response change over a person’s lifetime	False	100 (16.2; 13.4–19.4)	297 (48.1; 44.1–52.2)	220 (35.7; 31.9–39.6)
The package insert for warfarin includes a warning about altered metabolism in individuals who have specific genetic variants	True	310 (50.2; 46.2–54.2)	73 (11.8; 9.4–14.7)	234 (38.0; 34.1–41.9)
Pharmacogenetic testing is currently available for most medications	False	189 (30.6; 27.1–34.5)	109 (17.7; 14.8–21.0)	319 (51.7; 47.7–55.7)
Pharmacogenetics has an important role in individualizing response to medications	True	453 (73.4; 69.7–76.8)	28 (4.5; 3.0–6.6)	136 (22.1; 18.9–25.6)
Pharmacogenetics has an important role in identifying drug-drug interactions.	True	359 (58.2; 54.2–62.1)	60 (9.7; 7.6–12.4)	198 (32.1;28.5–36.0)

### Respondents’ perceptions towards pharmacogenetics and its implications

The overall mean (SD) perceptions score of respondents was 4.2 (1.8) [positive perception]. Over two-thirds (n = 433; 70.2%; 95% CI: 66.4–73.4) of participants expressed an overall positive perceptions of ≥ 4.0. Table 3 shows the distribution of participants’ responses to the six items used to assess perceptions towards pharmacogenetics and its implications.

**Table 3:** Respondents’ perceptions towards pharmacogenetics and its implications (n = 617).

Responses the perception items	Pharmacists (n = 238) Frequency (%)	Physicians (n = 379) Frequency (%)	Total (n = 617) Frequency (%)	P value
1.Pharmacogenetics is relevant to my current clinical practice.				
Agree	164 (69.0)	240 (63.3)	404 (65.5)	0.027*
Neutral	55 (23.0)	121 (32.0)	176 (28.5)	
Disagree	19 (8.0)	18 (4.7)	(6.0)	
2. Pharmacists should be required to have some knowledge of pharmacogenetics.				
Agree	214 (90.0)	326 (86.0)	540 (87.5)	0.303
Neutral	19 (8.0)	45 (12.0)	64 (10.4)	
Disagree	5 (2.0)	8 (2.0)	2.1)	
3. Pharmacogenetic testing should be applied into my clinical practice.				
Agree	180 (75.6)	230 (60.7)	410 (66.5)	<0.001*
Neutral	50 (21.0)	137 (36.1)	187 (30.3)	
Disagree	8 (3.4)	12 (3.2)	3.2)	
4. Pharmacists should be asked by healthcare professionals for recommendations on appropriate use of pharmacogenetic testing.				
Agree	162 (68.1)	259 (68.3)	421 (68.2)	0.923
Neutral	63 (26.5)	102 (27.0)	165 (26.7)	
Disagree	13 (5.5)	18 (4.7)	5)	
5. I should be able to provide information on appropriate use of pharmacogenetic testing.				
Agree	177 (74.4)	224 (59.1)	401 (65.0)	<0.001*
Neutral	43 (18.1)	137 (36.1)	180 (29.2)	
Disagree	(7.6)	18 (4.7)	(5.8)	
6. Pharmacogenetics will improve our ability to more effectively control drug therapy expenditures.				
Agree	184 (77.3)	225 (59.4)	409 (66.3)	<0.001*
Neutral	40 (16.8)	131 (34.6)	171 (27.7)	
Disagree	14 (5.9)	23 (6.1)	37 (6.0)	

*Significant difference between physicians and pharmacists using Chi-square test

About two-thirds of respondents expressed positive perceptions towards the relevance of pharmacogenetics to their current clinical practice (n = 404; 65.5%; 95% CI: 61.6, 69.2), the application of pharmacogenetic testing into their clinical practice (n = 410; 66.5%; 95% CI: 62.6–70.1), the suggestion that they should be capable to provide information on the appropriate use of pharmacogenetic testing (n = 401; 65.0%; 95% CI: 61.1–68.7), that healthcare providers should ask pharmacists for recommendations on appropriate use of pharmacogenetic testing (n = 421; 68.2%; 95% CI: 64.4–71.9), and that pharmacogenetics would improve their ability to more effectively control drug therapy expenditures (n = 409; 66.3%; 95% CI: 62.4–70.0). Over four-fifths (n = 540; 87.5%; 95% CI: 84.6–90.0) of the respondents agreed that pharmacists should be required to have some knowledge of pharmacogenetics. Pharmacists expressed significantly more positive attitudes than physicians towards the relevance of pharmacogenetics and its application into their clinical practice, the suggestion that they should be capable to provide information on appropriate use of pharmacogenetic testing, and the role of pharmacogenetics on controlling drug therapy expenditures (p<0.05).

### Respondents’ confidence in applying pharmacogenetics in their practice settings

Respondents’ overall mean (SD) confidence score was 1.0 (1.3) [low confidence]. Only 16.0% (n = 99; 95% CI: 13.3–19.2) of respondents claimed overall high self-confidence scores of ≥ 3.0. Table 4 shows the distribution of the participants’ responses to the four items used to assess their confidence in applying pharmacogenetics in their practice settings. Less than one-third of participants agreed that they were able to identify reliable sources of pharmacogenetic information for healthcare providers and patients (n = 198; 32.1%; 95% CI: 28.5–36.0), able to accurately apply the results of a pharmacogenetic test to drug therapy selection, dosing, or monitoring (n = 165; 26.7%; 95% CI: 23.3–30.5), and capable of identifying drugs that require pharmacogenetic testing (n = 145; 23.5%; 95% CI: 20.3–27.1), Less than one-fifth (n = 115; 18.6%; 95% CI: 15.7–22.0) agreed that they were readily able to determine what pharmacogenetic tests are available within their healthcare system. Pharmacists expressed significantly higher self-confidence than physicians in identifying reliable sources of information regarding pharmacogenetics for healthcare professionals and patients (P<0.05).

**Table 4:** Respondents’ confidence in applying pharmacogenetics in their practice settings (n = 617).

Responses to the self-confidence items	Pharmacists (n = 238) Frequency (%)	Physicians (n = 379) Frequency (%)	Total (n = 617) Frequency (%)	P value
1. I can identify drugs that need pharmacogenetic testing.				
Agree	59 (24.8)	86 (22.7)	145 (23.5)	0.765
Neutral	104 (43.7)	176 (46.4)	280 (45.4)	
Disagree	75 (31.5)	117 (30.9)	1.1)	
2. I can identify reliable sources of information regarding pharmacogenetics for healthcare professionals and patients.				
Agree	96 (40.3)	102 (26.9)	198 (32.1)	0.002*
Neutral	85 (35.7)	166 (43.8)	251 (40.7)	
Disagree	57 (23.9)	111 (29.3)	7.2)	
3. I can readily determine the available pharmacogenetic tests within our healthcare system.				
Agree	44 (18.5)	71 (18.7)	115 (18.6)	0.899
Neutral	96 (40.3)	146 (38.5)	242 (39.2)	
Disagree	98 (41.2)	162 (42.7)	2.1)	
4. I can accurately apply the results of a pharmacogenetic test to drug therapy selection, dosing, or monitoring.				
Agree	64 (26.9)	101 (26.6)	165 (26.7)	0.973
Neutral	97 (40.8)	152 (40.1)	249 (40.4)	
Disagree	77 (32.4)	126 (33.2)	203 (32.9)	

*Significant difference between physicians and pharmacists using Chi-square test

### Factors influencing knowledge, perceptions and confidence

Table 5 presents the results on the influence of the respondents’ characteristics on their knowledge, perceptions, and self-confidence towards pharmacogenetics. Profession, age, gender and previous pharmacogenetics training or education were found not to significantly influence the level of knowledge (p>0.05). However, those who had ≥ 10 years of practice experience scored significantly better levels of knowledge than did those with ≤ 10 years of experience [48.0% (25.4) vs. 43.6% (23.3); p = 0.002]. Profession and pharmacogenetic training were different with respect to overall perceptions. Positive overall perceptions towards pharmacogenetics and its implications was found to be significantly common among pharmacists, and those who had previous pharmacogenetic training or education. Practice experience and pharmacogenetics training or education were different with respect to overall self-confidence. Overall self-confidence was found to be significantly common among those who had practice experience of ≥ 10 years, and those who had previous pharmacogenetics training or education.

**Table 5:** Influence of respondents’ characteristics on their level of knowledge, perceptions, and self-confidence towards pharmacogenetics (n = 617).

Variable	Overall mean (SD) percentage knowledge score (%)	P value	Overall mean (SD) Perceptions score	P value	Overall mean (SD) self-confidence score	P value
***Profession***						
Pharmacist	48.8 (22.7)	0.181	4.5 (1.7)	<0.001*	1.1 (1.3)	0.082
Physician	43.4 (25.4)		3.9 (1.9)		1.0 (1.3)	
***Age***						
≤35	44.4 (23.3)	0.073	4.1 (1.9)	0.185	1.0 (1.3)	0.094
≥36	47.8 (25.9)		4.3 (1.7)		1.1 1.3)	
***Gender***						
Male	45.7 (25.9)	0.440	4.1 (1.8)	0.07	1.0 (1.2)	0.061
Female	45.7 (22.3)		4.3 (1.8)		1.1 (1.3)	
***Work Experience***						
<10	43.6 (23.3)	0.002*	4.0 (1.9)	0.067	0.9 (1.3)	0.013*
≥10	48.0 (25.4)		4.3 (1.7)		1.1 (1.3)	
***Attended pharmacogenetics training or education***						
No	45.7 (25)	0.970	4.1 (1.8)	0.016*	0.9 (1.2)	<0.001*
Yes	46.0 (22)		4.7 (1.8)		2.0 1.6)	

*Significant difference between the two groups using Mann-Whitney test

### Respondents’ educational format preferences

**Fig 1:**
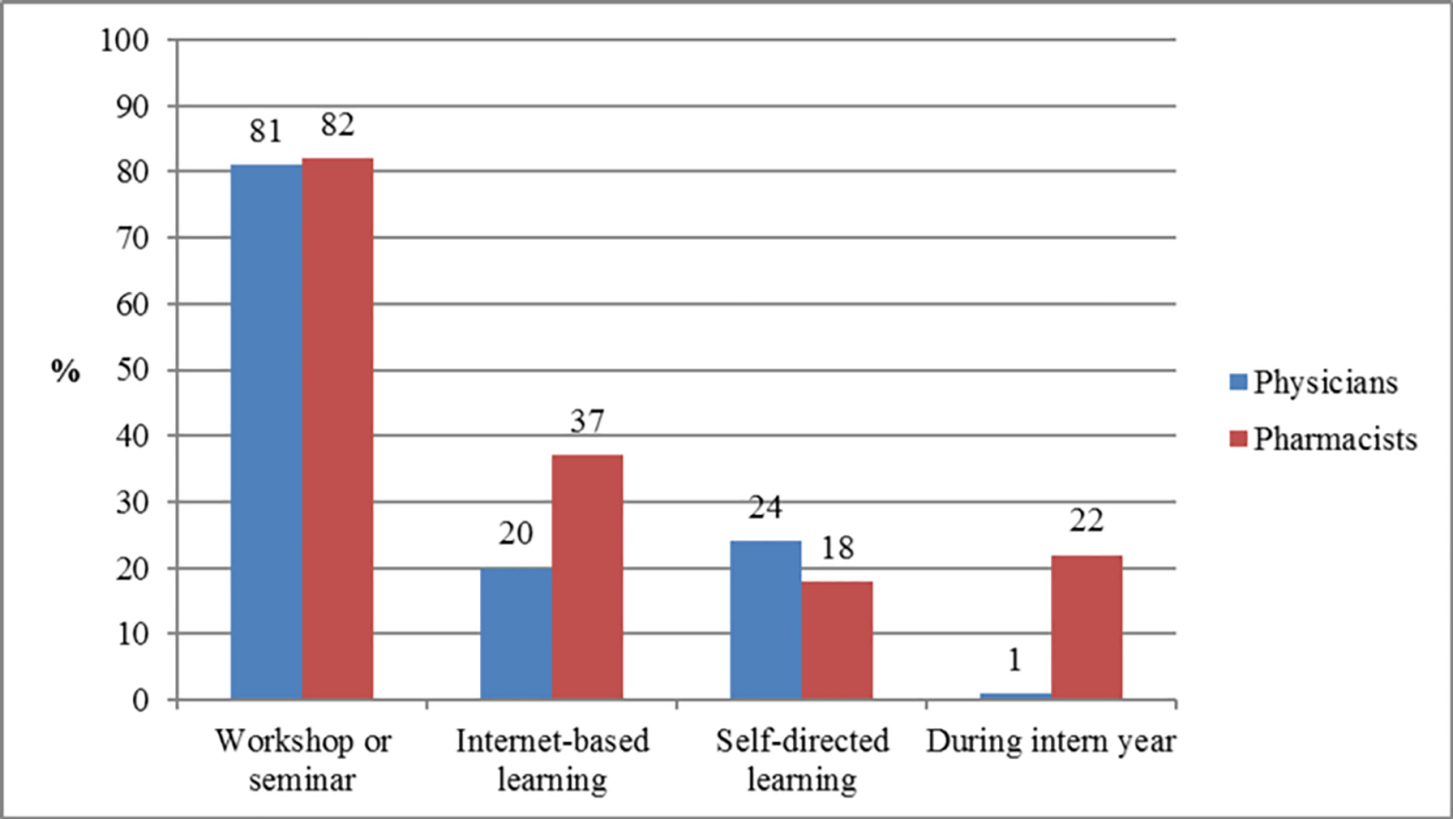
Respondents’ educational format preferences in pharmacogenetics.

The top preferred educational format in pharmacogenetics was workshops or seminars (n = 501; 81.2%; 95% CI: 77.8–84.2), followed by internet-based learning (n = 166; 26.9%: 95% CI: 23.5–30.6), self-directed learning (n = 134; 21.7%; 95% CI: 18.6–25.2), and learning during the internship period (n = 56; 9.1%; 95% CI: 7.0–11.7). Fig 1 presents the distribution of the physicians’ and pharmacists’ responses regarding their educational format preferences.

### Perceived barriers to the application of pharmacogenetic testing

**Fig 2:**
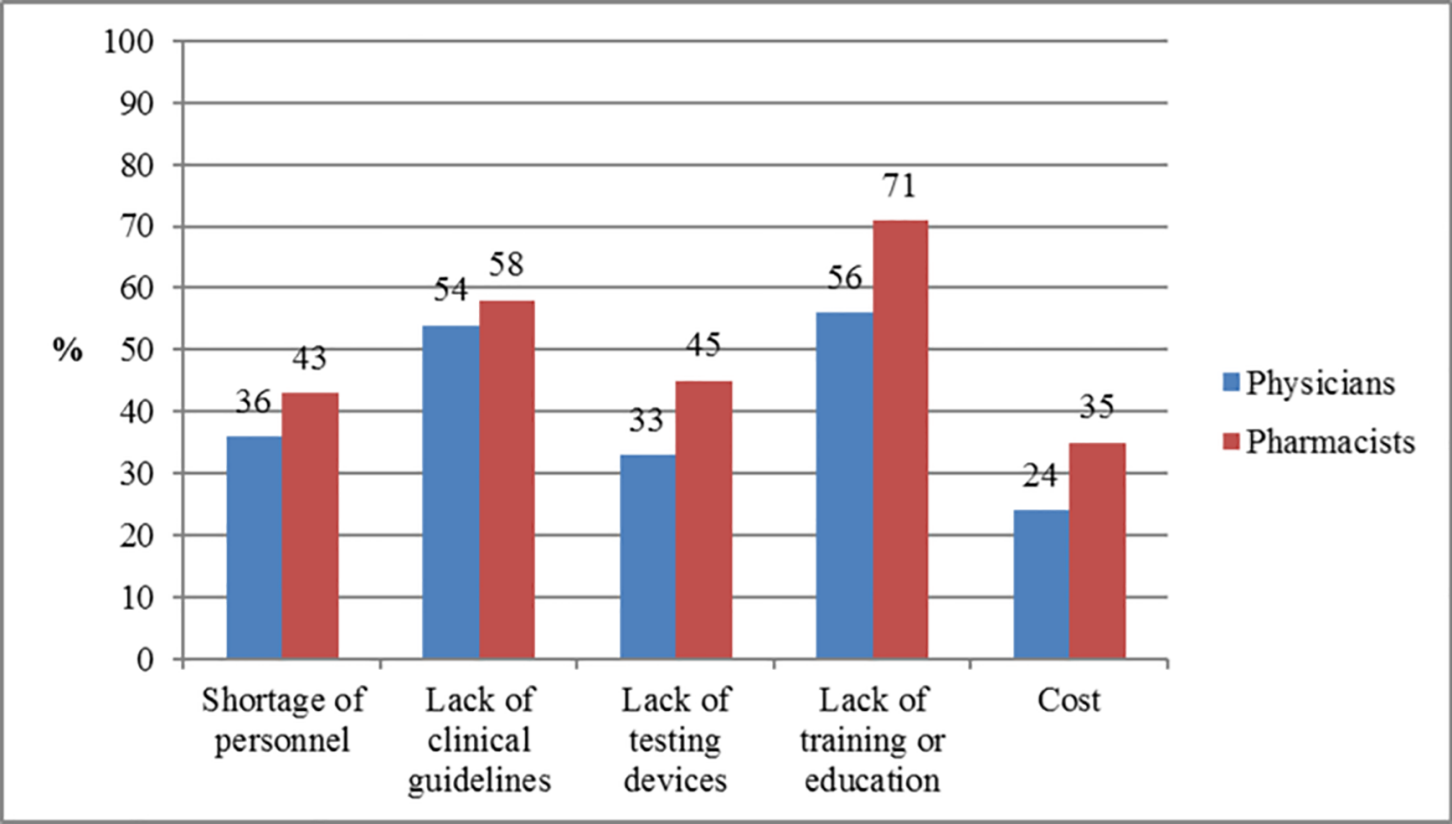
Perceived barriers facing the implementation of pharmacogenetic testing in clinical practice.

The top two perceived barriers facing the implementation of pharmacogenetic testing in respondents’ clinical practice were lack of training or education in pharmacogenetics (n = 380; 61.6%; 95% CI: 57.6–65.4) and lack of clinical guidelines on pharmacogenetics practice (n = 342; 55.4%; 95% CI: 51.4–59.4). Other barriers included were shortage of personnel (n = 239; 38.7%: 95% CI: 34.9–42.7), lack of testing devices (n = 234; 37.9%; 95% CI: 34.1–41.9), and cost of the testing devices (n = 175; 28.4%; 95% CI: 24.8–32.1). Fig 2 presents the distribution of the physicians’ and pharmacists’ responses regarding the barriers facing the application of pharmacogenetic testing in their practice settings.

## Discussion

This is the first known study to be performed in Kuwait, and the second in the Middle Eastern region to demonstrate the current level of physicians’ and pharmacists’ knowledge, perceptions and confidence towards pharmacogenetics and its clinical implications. The present findings contribute to the limited amount of existing literature in the developing countries about these outcomes, and allow for important comparative work with existing and future investigations in middle-eastern countries, and worldwide. The current results highlight important concerns and reveal a baseline quantitative data-set that will aid in the assessment of current pharmacogenetic practice in Kuwait, and could be utilised by policy-makers in educational and healthcare systems to design future targeted multifaceted interventions to promote the implementation of pharmacogenetic testing within clinical practice settings in Kuwait.

In the present study, lower percentages of participants were exposed to pharmacogenetic education or training (8.9%), applied pharmacogenetic testing in their clinical practice (9.4%), and provided patient counselling on the results of pharmacogenetic testing (9.1%), over 90% of whom were physicians. These results are distinct from those reported in a prior study from Malaysia in which about half of the respondents indicated prior exposure to education in pharmacogenetics, physicians were less exposed than pharmacists, and only 5.8% of respondents applied the testing in their practice [28]. Low use of pharmacogenetic testing (12.9%) and inadequate information about pharmacogenetic testing (89.7%) were also reported by another study in the USA [20]. However, other studies from the USA reported higher percentages of physicians ordering pharmacogenetic testing in their practice, ranging from 44% to 74% [21,22]. Our findings that most of those who had been exposed to pharmacogenetic education and applied pharmacogenetic testing and patient counselling in their practice were physicians could partly be explained by the fact that the physicians in the Middle Eastern region are the leaders in all clinical decisions and are responsible for the implementation of pharmacogenetics into their practice settings [24]. Furthermore, clinical pharmacy practice in Kuwait is still under development [29]. These findings highlight the need for more effective education of physicians and pharmacists on pharmacogenetics and the clinical value of the application of pharmacogenetic testing in their practice.

The study participants expressed low knowledge regarding pharmacogenetics and pharmacogenetic testing, which is almost consistent with findings from previous reports evaluating knowledge among physicians and pharmacists [16,20,24,28]. In contrast, higher knowledge (ranging from 53% to 89%) was reported by prior studies from the USA during the period from 2010 to 2013 [16]. The present findings revealed no significant difference in the level of knowledge among physicians and pharmacists: this is similar to that reported in Qatar [24]. However, study participants with practice experience of ≥ 10 years had significantly better levels of knowledge than did those with ≤ 10 years of experience. In contrast, a study from Malaysia reported higher knowledge scores among pharmacists compared to physicians and those with fewer years of practice experience [28]. Most of the respondents indicated that genetic determinants of drug response change over a person’s lifetime, which is a common misconception that needs to be corrected. The current results underscore the urgent need for effective educational programs on pharmacogenetics and pharmacogenetic testing at the undergraduate and postgraduate levels to accelerate the implementation of pharmacogenetic testing in the Kuwaiti healthcare system. This is confirmed by the fact that most of the respondents indicated that lack of education or training as the top perceived barrier facing the implementation of pharmacogenetic testing in their clinical practice. This important challenge can be overcome through effective collaboration between medical and pharmacy schools, the Ministry of Health and the professional organizations to update the current undergraduate curricula to improve the pharmacogenetics knowledge base of the students. It is the responsibility of the faculties of medicine and pharmacy to prepare physicians and pharmacists entering clinical practice to effectively use genetic data in the delivery of precision medicine. The pharmacogenomics education and implementation should be incorporated in the clinical phases of the undergraduate curricula over the last three years of the Faculty of Medicine and the last two years of the Faculty of Pharmacy. Also, it is equally essential that they develop and implement appropriate continuous professional education programs to healthcare providers through regular workshops and seminars, since these were the most preferred educational formats by most of the respondents. In addition, instructional software to improve understanding and application of pharmacogenetics can be applied to online training, since it was the second preferred educational format in this study [30].

Another important barrier that needs to be overcome is the lack of clinical guidelines on pharmacogenetic practice. This finding emphasizes that the future training of healthcare professionals should include a focus on the use of resources for evidence-based application of pharmacogenetics, such as the Clinical Pharmacogenetics Implementation Consortium (CPIC) guidelines [31].

Despite the reported knowledge gap on pharmacogenetics among the study participants, over two-thirds of them expressed overall positive perceptions towards pharmacogenetics and its implications, in accordance with the results in similar previous studies [16,19,24,28]. This positive perception can be attributed to the expected benefits from pharmacogenetics through personalized medicine and emphasizes that pharmacogenetics should be an integral part of the healthcare system. Also, it may indicate that pharmacogenetics practice has the potential to be implemented by the respondents.

Despite the similar level of knowledge on pharmacogenetics among the physicians and pharmacists in the present study, pharmacists expressed significantly more positive attitudes than physicians towards the relevance of pharmacogenetics and its application into their clinical practice, being capable to provide information on appropriate use of pharmacogenetic testing, and the role of pharmacogenetics in controlling drug therapy expenditures. These findings are in agreement with those reported in Qatar and Malaysia [24,28]. These results support the emerging and expanding role of pharmacists in delivering personalized pharmacotherapy services and are of importance to their expected leadership role in pharmacogenetic services. This is supported by the fact that over two-thirds of the physicians in this study agreed that pharmacists should be required to have some knowledge of pharmacogenetics (86%) and that healthcare providers should ask pharmacists for recommendations on appropriate use of pharmacogenetic testing (68.3%). Previous studies reveal that other healthcare professionals, including physicians have supported the key role that pharmacists can play in providing pharmacogenetic services, which should be led by pharmacists rather than physicians [23,32,33].

In the present study, respondents indicated a low level of overall self-confidence towards implementation of pharmacogenetics in their practice settings. About seven in ten respondents were either unsure or could not identify drugs that require pharmacogenetic testing, identify reliable sources of information about pharmacogenetics, or apply the results of pharmacogenetic testing to drug selection, dosing or monitoring. This is in concordance with similar previous reports [16,19,23,28],and could indicate the low level of recognition among the study participants of the FDA labelling information/recommendations as standard practice. This lack of self-confidence may stem from the deficiency in their knowledge on pharmacogenetics and highlights the need for appropriate and effective educational programmes, such as those being successfully implemented for healthcare students and practitioners in other countries [34–37]. Pharmacists expressed significantly higher self-confidence than physicians in identifying reliable sources of information regarding pharmacogenetics for healthcare professionals and patients. This finding, in addition to their highly positive perceptions, suggests that pharmacists may have great interest and willingness to serve many active roles in the implementation of pharmacogenetics in the healthcare setting to ensure optimal individualized medication therapy for appropriate patients.

The present findings reveal that respondents who had attended previous pharmacogenetic training or education had similar levels of knowledge, but positive perceptions and high confidence towards pharmacogenetics and its implications, compared to those who had not attended previous training. This is in contrast with the findings of a prior study, which reported that the previous pharmacogenetic training directly impacted healthcare providers’ knowledge [20]. It has been reported that healthcare providers with younger age of ≤ 36 years and those who had been practicing for ≤ 4 years had the highest knowledge and the most positive attitude towards pharmacogenetics [28]. Arguably, in the current study, physicians and pharmacists with more practice experience showed significantly more knowledge and greater self-confidence than did those with less experience. A possible explanation for this result could be the deficient education about pharmacogenetics and its clinical value in the curricula of medical and pharmacy schools, which may have disadvantaged recent graduates. Furthermore, practitioners with more experience may have been exposed to more discussions about pharmacogenetics and its implications during their longer practice experience. A previous study in Malaysia indicated that lack of knowledge on pharmacogenetics is more prevalent among males compared to females [28]. In the present study, there was no significant difference in the level of knowledge among males and females. Hence, these findings highlight the need for further qualitative research to provide better understanding of the predictors of knowledge, perceptions and self-confidence among healthcare professionals in Kuwait.

### Strengths and limitations

The strengths of this study included the high response rate, which could indicate the importance of this topic to physicians and pharmacists and that they were interested to share their opinions. A further strength was the adequate sample size and sampling method to produce representative data regarding the study population; therefore, the present findings can be generalised to healthcare providers in secondary healthcare settings in Kuwait. In addition, this study fills a gap in the limited existing literature in the developing countries and provides useful information for healthcare providers’ knowledge, perceptions and confidence towards pharmacogenetics and its implications in the Middle Eastern region. Limitations of this study include the fact that the study population was selected from secondary healthcare settings, as responses from healthcare providers in primary and tertiary settings may be different. Hence, the current results may not be representative of all healthcare providers in Kuwait. In addition, a further weakness is the use of a Likert scale in the survey, which is prone to central tendency bias (selecting ‘neutral’ or ‘unsure’ answers). Respondents who are honestly uncertain about or unfamiliar with the topic may select the middle option. Despite the presence of this middle option, more than half of participants either agreed or disagreed with most of the questionnaire items, minimising the potential central tendency bias. A further limitation is the social desirability bias: respondents might have offered favourable answers to conform to the more socially accepted view. Also, the cross-sectional nature of the survey represented one point in time and, therefore, does not reflect any changes in respondents’ knowledge, perceptions, and confidence over time regarding pharmacogenetics and its implications.

## Conclusions

The present findings reveal that despite the physicians’ and pharmacists’ low levels of knowledge, confidence, and practice towards pharmacogenetics, they have positive perceptions of pharmacogenetics and its clinical implications. The positive perceptions demonstrated by respondents may emphasize that pharmacogenetics should be an integral part of the healthcare system in Kuwait and also may indicate that pharmacogenetic practice has the potential to be implemented by the respondents, particularly pharmacists. Therefore, there is an urgent need for education and training in the area of pharmacogenetics and its clinical implications through continuous professional development programs and its integration into medical and pharmacy schools’ curricula. The current results show a general agreement that pharmacists should be required to have knowledge of pharmacogenetics and should be asked by healthcare professionals for recommendations on appropriate use of pharmacogenetic testing. Furthermore, pharmacists expressed higher levels of self-confidence than physicians in identifying reliable sources of information regarding pharmacogenetics for healthcare professionals and patients. This will allow pharmacists to play a crucial role in influencing the future of pharmacogenetics in Kuwait. Lack of education and clinical guidelines are the major barriers perceived by respondents towards the implementation of pharmacogenetics, followed by shortage of personnel, lack of testing devices and the high cost of such devices. Policy makers in educational and healthcare systems need to collaborate and focus on alleviating those identified barriers towards the implementation of pharmacogenetics into clinical practice.

## Supporting information

S1 Text Albassam-PLOS ONE-SPSS raw data. (SAV)

S2 Text Albassam-PLOS ONE-Questionnaire. (DOCX)
