# Correction: Knowledge, perceptions and confidence of physicians and pharmacists towards pharmacogenetics practice in Kuwait

**DOI:** 10.1371/journal.pone.0212118

**Published:** 2019-02-07

**Authors:** The PLOS ONE staff

In Table 3, the Total Frequency of respondents who answered Disagree to perception item 1–5 is partially missing. Additionally in Table 4, the Total Frequency of respondents who answered Disagree to perception item 1–3 is partially missing. There are minor formatting errors in Table 5. Please see the corrected Tables 3–5 here. The publisher apologizes for the errors.

**Table 3:** Respondents’ perceptions towards pharmacogenetics and its implications (n = 617).

Responses the perception items	Pharmacists (n = 238) Frequency (%)	Physicians (n = 379) Frequency (%)	Total (n = 617) Frequency (%)	P value
1. Pharmacogenetics is relevant to my current clinical practice.				
Agree	164 (69.0)	240 (63.3)	404 (65.5)	0.027*
Neutral	55 (23.0)	121 (32.0)	176 (28.5)	
Disagree	19 (8.0)	18 (4.7)	37 (6.0)	
2. Pharmacists should be required to have some knowledge of pharmacogenetics.				
Agree	214 (90.0)	326 (86.0)	540 (87.5)	0.303
Neutral	19 (8.0)	45 (12.0)	64 (10.4)	
Disagree	5 (2.0)	8 (2.0)	13 (2.1)	
3. Pharmacogenetic testing should be applied into my clinical practice.				
Agree	180 (75.6)	230 (60.7)	410 (66.5)	<0.001*
Neutral	50 (21.0)	137 (36.1)	187 (30.3)	
Disagree	8 (3.4)	12 (3.2)	20 (3.2)	
4. Pharmacists should be asked by healthcare professionals for recommendations on appropriate use of pharmacogenetic testing.				
Agree	162 (68.1)	259 (68.3)	421 (68.2)	0.923
Neutral	63 (26.5)	102 (27.0)	165 (26.7)	
Disagree	13 (5.5)	18 (4.7)	31 (5.0)	
5. I should be able to provide information on appropriate use of pharmacogenetic testing.				
Agree	177 (74.4)	224 (59.1)	401 (65.0)	<0.001*
Neutral	43 (18.1)	137 (36.1)	180 (29.2)	
Disagree	18 (7.6)	18 (4.7)	36 (5.8)	
6. Pharmacogenetics will improve our ability to more effectively control drug therapy expenditures.				
Agree	184 (77.3)	225 (59.4)	409 (66.3)	<0.001*
Neutral	40 (16.8)	131 (34.6)	171 (27.7)	
Disagree	14 (5.9)	23 (6.1)	37 (6.0)	

*Significant difference between physicians and pharmacists using Chi-square test

**Table 4:** Respondents’ confidence in applying pharmacogenetics in their practice settings (n = 617).

Responses to the self-confidence items	Pharmacists (n = 238) Frequency (%)	Physicians (n = 379) Frequency (%)	Total (n = 617) Frequency (%)	P value
1. I can identify drugs that need pharmacogenetic testing.				
Agree	59 (24.8)	86 (22.7)	145 (23.5)	0.765
Neutral	104 (43.7)	176 (46.4)	280 (45.4)	
Disagree	75 (31.5)	117 (30.9)	192 (31.1)	
2. I can identify reliable sources of information regarding pharmacogenetics for healthcare professionals and patients.				
Agree	96 (40.3)	102 (26.9)	198 (32.1)	0.002*
Neutral	85 (35.7)	166 (43.8)	251 (40.7)	
Disagree	57 (23.9)	111 (29.3)	168 (27.2)	
3. I can readily determine the available pharmacogenetic tests within our healthcare system.				
Agree	44 (18.5)	71 (18.7)	115 (18.6)	0.899
Neutral	96 (40.3)	146 (38.5)	242 (39.2)	
Disagree	98 (41.2)	162 (42.7)	260 (42.1)	
4. I can accurately apply the results of a pharmacogenetic test to drug therapy selection, dosing, or monitoring.				
Agree	64 (26.9)	101 (26.6)	165 (26.7)	0.973
Neutral	97 (40.8)	152 (40.1)	249 (40.4)	
Disagree	77 (32.4)	126 (33.2)	203 (32.9)	

*Significant difference between physicians and pharmacists using Chi-square test

**Table 5:** Influence of respondents’ characteristics on their level of knowledge, perceptions, and self-confidence towards pharmacogenetics (n = 617).

Variable	Overall mean (SD) percentage knowledge score (%)	P value	Overall mean (SD) Perceptions score	P value	Overall mean (SD) self-confidence score	P value
***Profession***						
Pharmacist	48.8 (22.7)	0.181	4.5 (1.7)	<0.001*	1.1 (1.3)	0.082
Physician	43.4 (25.4)		3.9 (1.9)		1.0 (1.3)	
***Age***						
≤35	44.4 (23.3)	0.073	4.1 (1.9)	0.185	1.0 (1.3)	0.094
≥36	47.8 (25.9)		4.3 (1.7)		1.1 (1.3)	
***Gender***						
Male	45.7 (25.9)	0.440	4.1 (1.8)	0.07	1.0 (1.2)	0.061
Female	45.7 (22.3)		4.3 (1.8)		1.1 (1.3)	
***Work Experience***						
<10	43.6 (23.3)	0.002*	4.0 (1.9)	0.067	0.9 (1.3)	0.013*
≥10	48.0 (25.4)		4.3 (1.7)		1.1 (1.3)	
***Attended pharmacogenetics training or education***						
No	45.7 (25)	0.970	4.1 (1.8)	0.016*	0.9 (1.2)	<0.001*
Yes	46.0 (22)		4.7 (1.8)		2.0 (1.6)	
