# Supplementary material for: Knowledge, perceptions and confidence of physicians and pharmacists towards pharmacogenetics practice in Kuwait
Source: PLoS One. 2018 Sep 5;13(9):e0203033. doi: 10.1371/journal.pone.0203033 (PMC6124749; doi:10.1371/journal.pone.0203033)
Supplement: S2 Text — (DOCX) [file pone.0203033.s002.docx]

**Questionnaire to determine knowledge, perceptions and confidence of physicians and pharmacists towards pharmacogenetics**

**A. Demographic and other Characteristics**

**Please fill in OR TICK (√) THE APPROPRIATE ANSWER**

1. **Gender:** Male Female
2. **Age (in years**): ……………
3. **Professional experience (in years):** **……………**
4. **Hospital Name:** ……..**…………………………..**
5. **What is your profession and current level of practice?**

**Physician**

Resident

Specialist

Consultant

Other (please specify) ……..**……………………..**

**Pharmacist**

Outpatient dispensing pharmacist

Inpatient dispensing pharmacist

Clinical pharmacist

Others (please specify) ……..**……………………..**

1. **Do you have any extra credentials (postgraduate study) apart from your bachelor’s degree?**

Yes No **If yes, please specify** ……..**……………………………………………….**

**B. Pharmacogenetic training/education and application in practice**

1. **Have you completed pharmacogenetic testing related training or education?**  Yes No
2. **Have you applied pharmacogenetic testing to drug therapy selection, dosing and monitoring for a patient in your practice setting?** Yes No
3. **Have you counseled patients on the results of their pharmacogenomics testing in your practice setting?** Yes No

**C. General knowledge on pharmacogenetics**

**Please insert tick (√) in the corresponding box that MOST appropriately reflects your answer.**

| **Statements** | **Answer** |
| --- | --- |
| **10. Genetic determinants of drug response change over a person’s lifetime.** | True False Do not know/Not sure |
| **11. The package insert for warfarin includes a warning about altered metabolism in individuals who have specific genetic variants.** | True False Do not know/Not sure |
| **12. Pharmacogenetic testing is currently available for most medications.** | True False Do not know/Not sure |
| **13. Pharmacogenetics has an important role in individualizing response to medications.** | True False Do not know/Not sure |
| **14. Pharmacogenetics has an important role in identifying drug-drug interactions.** | True False Do not know/Not sure |

**D. Perceptions towards pharmacogenetics and its implications**

**Please insert tick (√) in the corresponding box that BEST fits your level of agreement with each statement.**

| **Statements** | **Agree** | **Neutral** | **Disagree** |
| --- | --- | --- | --- |
| **15. Pharmacogenetics is relevant to my current clinical practice.** |  |  |  |
| **16. Pharmacists should be required to have some knowledge of pharmacogenetics.** |  |  |  |
| **17. Pharmacogenetic testing should be applied into my clinical practice.** |  |  |  |
| **18. Pharmacists should be asked by healthcare professionals for recommendations on appropriate use of pharmacogenetic testing.** |  |  |  |
| **19. I should be able to provide information on appropriate use**  **of pharmacogenetic testing.** |  |  |  |
| **20. Pharmacogenetics will improve our ability to more effectively control drug therapy expenditures.** |  |  |  |
| **E. Confidence in applying pharmacogenetics in your practice settings**  **Please insert tick (√) in the corresponding box that BEST fits your level of agreement with each statement.** | | | |
| **Statements** | **Agree** | **Neutral** | **Disagree** |
| **21. I can identify drugs that need pharmacogenetic testing.** |  |  |  |
| **22. I can identify reliable sources of information regarding pharmacogenetics for healthcare professionals and patients.** |  |  |  |
| **23. I can readily determine the available pharmacogenetic tests within our healthcare system.** |  |  |  |
| **24. I can accurately apply the results of a pharmacogenetic test to drug therapy selection, dosing, or monitoring.** |  |  |  |

**F. Future education about pharmacogenetics**

**25. Which type of education do you prefer to learn about pharmacogenetics? Please tick (√) all that apply.**

Workshops or seminars

Internet based learning activities

Self-directed learning

During internship year

Others (Please specify): ……**………………………………………………………………………**

**G. Barriers to the application of pharmacogenetic testing**

**26. Which of the following do you think are barriers for the implementation of pharmacogenetic testing in your practice setting? Please tick (√) all that apply.**

Shortage of personnel

Lack of clinical guidelines on pharmacogenetic practice

Lack of testing devices

Lack of training or education

Cost of the testing devices

Others (Please specify): **………………………………………………………………………………**
